# Hepatoprotective action of *Dendrobium officinale* polysaccharide against MAFLD: modulation of gut microbiota-metabolite cascade controls TLR4/NF-κB inflammatory response

**DOI:** 10.3389/fphar.2026.1953606

**Published:** 2026-09-24

**Authors:** Yumei Xu, Yanmei Zhang, Shuqing Li, Heyan Yao, Yang Yang, Cuiping Yu, Shunzhen Zhang

**Affiliations:** 1 School of Chinese Materia Medica, Yunnan University of Chinese Medicine, Kunming, Yunnan, China; 2 The Third Affiliated Hospital of Yunnan University of Chinese Medicine, Kunming, Yunnan, China

**Keywords:** *Dendrobium officinale* polysaccharide, intestinal microflora-derived metabolites, lipopolysaccharide, medicinal and edible homologous plant, metabolic dysfunction-associated fatty liver disease, TLR4/NF-κB inflammatory pathway

## Abstract

**Introduction:**

Metabolic dysfunction-associated fatty liver disease (MAFLD) threatens public health worldwide, and *Dendrobium officinale* polysaccharide (DOP) exhibits promising hepatoprotective potential, yet its regulatory mechanism targeting the gut microbiota-metabolite-TLR4/NF-κB inflammatory axis remains poorly defined.

**Methods:**

In this study, *in-vivo* rat and *in-vitro* HepG2 cell MAFLD models were established. Male Sprague-Dawley (SD) rats received 12-week high-fat diet (HFD) feeding to induce MAFLD; HepG2 cells were exposed to a 1 mmol/L free fatty acid (FFA) mixture (oleic acid/palmitic acid = 2:1) for 24 h to trigger cellular steatosis. Serum, hepatic and cellular biochemical indexes, intestinal permeability factors and inflammatory mediators were detected. Hepatic pathological lesions and lipid accumulation were evaluated via hematoxylin-eosin (HE) and Oil Red O staining. Fecal metagenomics and untargeted metabolomics were applied to profile gut microbes and differential metabolites. RT-qPCR and Western blotting were used to assess tight-junction protein expression and TLR4/NF-κB pathway activation.

**Results:**

DOP markedly alleviated hepatic lipid accumulation, restored liver function abnormalities and mitigated hepatic pathological damage. DOP reshaped the disturbed gut flora: it reduced lipopolysaccharide (LPS)-enriched Gram-negative bacteria, elevated short-chain fatty acid-producing probiotics, modified intestinal metabolites, repaired the intestinal barrier and prevented intestinal LPS leakage. Both cellular and animal experiments demonstrated that DOP down-regulated TLR4, MyD88 and NF-κB p65 levels, increased IκBα expression and suppressed downstream pro-inflammatory mediators.

**Discussion:**

Collectively, DOP confers protective effects against MAFLD through remodeling gut microbiota and intestinal metabolites, preserving intestinal-barrier integrity, and inhibiting LPS-provoked hepatic TLR4/NF-κB inflammatory activation.

## 1 Introduction

MAFLD represents a chronic progressive liver disorder driven by overnutrition and insulin resistance in genetically predisposed individuals (Habibullah et al., 2025). Recent international consensus has substantially updated diagnostic criteria for fatty liver disease. Distinct from the previous NAFLD definition relying on exclusion of alcohol intake, the MAFLD diagnostic framework adopts an inclusion standard: hepatic steatosis accompanied by metabolic dysfunction, as formally endorsed by the 2025 Asian Pacific Association for the Study of the Liver (APASL) clinical practice guidelines (Eslam et al., 2025). This shift better highlights the central role of metabolic disturbance in disease pathogenesis. Modern medicine has achieved partial progress in MAFLD prevention and treatment; nevertheless, the clinical application of conventional western medicines is restricted by potential hepatotoxicity and additional metabolic burden. In contrast, natural edible plant-derived functional ingredients exert multi-component and multi-target regulatory effects. A wide range of phytochemicals from edible plants have been verified to alleviate glycolipid metabolic disturbance and hepatic steatosis (Yi et al., 2024). They possess unique advantages in dietary intervention to improve liver function, relieve inflammation and retard hepatic lesion progression, and have become a research hotspot for developing new functional foods against metabolic liver diseases (Balakina et al., 2025) Growing evidence underscores the tight crosstalk between obesity, gut homeostasis and MASLD pathogenesis (Basil et al., 2024; Benedé-Ubieto et al., 2024).

DO is a well-known Dao-di medicinal plant indigenous to Yunnan Province. Its dried stems have long been used to nourish yin, promote fluid secretion, and protect gastric health. As a typical representative of medicinal and edible homologous plants, DO embodies the integrated pharmacological and nutritional properties that are central to the theory of food–medicine homology (Cong, 2024). In accordance with the modern research framework of food–medicine homology, DO holds promising prospects for developing functional agents against metabolic liver diseases (Li et al., 2026). Mounting pharmacological research has demonstrated that DO contains abundant bioactive substances such as polysaccharides, flavonoids, and amino acids, which exhibit hepatoprotective, hypoglycemic, hypolipidemic, antioxidant, and immunomodulatory activities (Xu et al., 2022; Guo et al., 2025). In our earlier work, the ultrafine powder derived from DO was demonstrated to effectively combat metabolic dysfunction-associated steatohepatitis (MASH) while concurrently modulating gut microbial composition (Tian et al., 2023). Among its various functional components, *Dendrobium officinale* polysaccharide (DOP) serves as the predominant water-soluble bioactive constituent. Hardly digested by the host, DOP displays prominent prebiotic properties after being fermented by intestinal flora (Wu et al., 2023; Song et al., 2025). However, the precise mechanism by which DOP modulates the intestinal microecology and its metabolites through gut microbiota, thereby intervening in the development of MAFLD, remains largely unclear. To address this, we established *in vivo* and *in vitro* MAFLD models, combined fecal metagenomic sequencing with untargeted metabolomics to reveal the protective mechanism of DOP targeting gut microbiota-metabolite axis. This research provides experimental support for the high-value utilization of Yunnan characteristic DO and the development of novel hepatoprotective functional foods.

## 2 Materials and methods

### 2.1 Materials

#### 2.1.1 Experimental animals

Male SPF Sprague-Dawley rats were purchased from Sibefu (Beijing) Biotechnology Co., Ltd., with the animal production permit SCXK (Beijing) 2024-0001. These rats reached an age of 8 weeks and exhibited body weights ranging from 180 to 200 g. Over the whole experimental timeline, all rats were reared within the specific-pathogen-free housing unit operated by the Laboratory Animal Center at Yunnan University of Chinese Medicine. Constant control was maintained over ambient parameters: a steady thermal range of 22 °C–26 °C, relative humidity kept at 40%–60%, alongside a 12-h light-dark alternating cycle. Unlimited access to standard chow and drinking water was provided to all rats during the experimental period. The whole animal trial scheme received ethical examination and official authorization from the Experimental Animal Ethics Committee affiliated with Yunnan University of Chinese Medicine (Ethics Approval No. YNUTCM-XMSS-G-20250070). All procedures strictly followed the relevant ethical guidelines for research involving laboratory animals.

#### 2.1.2 Experimental cells and bacterial strains

All cellular functional assays were carried out using HepG2, a human hepatocellular carcinoma strain sourced from Wuhan Pricise Scientific Corporation (product catalog CL-010). Cells at the fifth passage, growing in the logarithmic phase with stable proliferation, were selected for subsequent assays. We obtained the *Lactobacillus acidophilus* strain for our cell-based experiments from the China General Microbiological Culture Collection Center (accession No. 1878).

#### 2.1.3 Experimental reagents and drugs

DOP used in animal experiments was obtained from Baoji Chenguang Biology (polysaccharide content ≥80%, Nos. HB20240914), while that used in cell experiments was purchased from Solarbio (UV-assayed purity ≥85%, Nos. SD9310). All sample aliquots were preserved in the Key Laboratory of Molecular Biology of Traditional Chinese Medicine, Kunming. Compound *Lactobacillus* acidophilus tablets (Yijunkang, Nos. H10940114, specification 5 × 10^6^ CFU/tablet) were employed as a positive control. For biochemical analysis, we employed commercial kits that measure TG, TC, HDL-C, LDL-C, ALT, AST, GGT, D-LA, and DAO, were all purchased from Nanjing Jiancheng Bioengineering Institute (Nos. A110-1-1, A111-1-1, A112-1-1, A113-1-1, C009-2-1, C010-2-1, C017-2-1, A019-3-1, and A088-3-1). Commercially available ELISA detection kits targeting LPS, IL-1β, IL-6 and TNF-α in animal-derived specimens were acquired from Jiangsu Meimian Industrial (Nos. MM-0647R1, MM-0047R1, MM-0190R1, MM-0180R1); corresponding kits for cell culture supernatants were also from Jiangsu Meimian (Nos. MM-1309H1, MM-0181H1, MM-0049H2, MM-0122H1). For protein analysis, a pre-stained protein marker (10-200 kDa, Servicebio, G2058) was used. Primary antibodies against TLR4, MyD88, IκBα, p-IκBα, p65, p-p65, and β-actin were sourced from ABclonal (Nos. A11226, A21905, A1187, AP0707, A2547, AP1294, AC026). Targeted immunoreagents detecting claudin-1 and occludin were supplied by HUABIO (Nos. HA721999, R150-33). HRP-tagged secondary goat anti-rabbit IgG (H + L) was procured via Beyotime (catalog A0208). Two chemical modulators were applied to probe molecular mechanisms: LPS, an agonist of Toll-like receptor 4 (TLR4), was obtained from Solarbio (L8880), while TAK-242, a selective TLR4 suppressor, was acquired from Shanghai Yuanye Bio-Technology (S80562). Other reagents included fetal bovine serum (ExCell Bio, FSP500), an oil red O staining kit specifically for cultured cells (Solarbio, G1262), oleic acid (Sigma, O7501), palmitic acid (Sigma, P0500), and bovine serum albumin (BSA, Solarbio, A8020). For bacterial culture, MRS broth and MRS agar were purchased from Solarbio (Nos. M8540, M8330), together with 2.5-L round-bottom vertical anaerobic culture bags and gas-generating packets (Hopebio, Nos. HBYY007, HBYY001).

#### 2.1.4 Experimental instruments

A range of specialized instruments was utilized to carry out the experimental procedures. Specifically, an ultra-micro spectrophotometer (Thermo Fisher, NanoDrop2000) was employed for nucleic acid quantification, while a gel imaging documentation system (Nanjing Jieda, 801) was used to visualize electrophoresis results. For gene amplification, a PCR thermal cycler (Eppendorf, 5332) was applied, followed by DNA sequencing performed on a DNBSEQ-T7 platform (BGI). Regarding metabolomics and lipid analysis, Chromatographic partitioning was accomplished via a Vanquish UHPLC platform (Thermo Fisher Scientific) fitted with a Hypersil Gold analytical column (100 × 2.1 mm, 1.9 μm). Subsequent MS profiling was implemented on a Q Exactive™ HF-X high-resolution mass spectrometer from the identical manufacturer. A microplate reader (Bioton Instruments Co., Ltd., SLXFA) was adopted for optical density measurements in biochemical assays. For protein analysis, a mini vertical electrophoresis tank (WIX, WIX-miniPRO4) and a four-gel mini transfer tank (WIX, WIX-miniBLOT4) were used to perform SDS-PAGE and Western blotting, respectively; protein signals were subsequently captured using an ultra-sensitive contact electronic chemiluminescence imager (e-BLOT, Touch Imager Pro). Morphological examination of tissue sections was facilitated by a slide scanning imaging system (TEKSQRAY, SQS-1000). Cell culture was maintained in a CO_2_ incubator (Shanghai Yiheng, BPN-1500H), and cell disruption was achieved with an ultrasonic disruptor (Shanghai Weimi, WM-1800T).

### 2.2 Methods

#### 2.2.1 Construction of MAFLD animal models, random animal allocation and therapeutic treatment regimens

*In vivo* experiment: to begin with, fifty male Sprague-Dawley rats of SPF grade underwent a 7-day acclimation period. We next randomly assigned them into several groups: a blank control (C), a model group (M), a positive control (YSJ), and three DOP treatment groups—low, medium, and high dose (DPL, DPM, DPH, respectively). Specifically, the model group consisted of ten rats (with two additional animals reserved for verifying successful model induction during the experiment), whereas each of the other groups included eight animals. In group C, We provided the rats with normal chow and distilled water. By comparison, the other experimental cohorts received high-fat chow over a 12-week timeline to establish pathological MAFLD phenotypes. Throughout this feeding period, the corresponding drug interventions were delivered via daily gavage. Regarding the dosages, the DOP groups received 250, 500, and 1,000 mg/(kg·d), respectively, while the YSJ group was given 270 mg/(kg·d). Compound *Lactobacillus acidophilus* tablets (YSJ) were selected as the positive control. Cumulative research demonstrates that *Lactobacillus acidophilus* improves gut microbiota homeostasis, suppresses hepatic lipid deposition and inflammatory response, and has been widely adopted as positive intervention agent in MAFLD animal studies (Wang et al., 2023; Lau et al., 2024). These dosage levels were established based on body surface area conversion and prior experimental findings. Ultimately, during the entire trial period, every rats had unrestricted access to standard chow and purified drinking water.

For cellular functional assays, HepG2 cultures at logarithmic growth phase were collected and resuspended to adjust the cell concentration to 5 × 10^5^ cells per milliliter. Subsequently, 1 mL aliquots of the resulting cell suspension were dispensed into every cavity of six-well culture plates. After 24 h of culture period to allow cell adherence, an MAFLD cellular model was generated by exposing all groups except the control (C) to 1.0 mM free fatty acids (FFA) for 24 h. Subsequently, the successfully modeled cells were separated into eight experimental categories: C, M, YSJ, DPL, DPM, DPH, LPS, and TAK-242. The intervention concentration for each drug was decided according to cell viability assay outcomes. Finally, all groups were treated with their respective agents for an additional 24-h period.

#### 2.2.2 Biochemical assays

We quantified the concentrations of TC, TG, HDL-C, LDL-C, ALT and AST within serum, hepatic tissue and cultured hepatocytes, alongside serum GGT, D-LA and DAO. All biochemical indices were assayed with standard commercial test kits following the official operating protocols supplied by each reagent vendor.

#### 2.2.3 Histological staining with hematoxylin-eosin (HE) and oil red O

Hepatic specimens underwent initial fixation using 4% paraformaldehyde solution. Subsequent tissue trimming was performed, followed by gradient ethanol dehydration steps. After paraffin embedding and sectioning, the slides were stained with HE and finally scanned for image capture. For Oil Red O staining, we collected and fixed additional liver samples, then dehydrated stepwise using 15% and 30% sucrose solutions, followed by frozen sectioning. After staining the sections with Oil Red O, we mounted them in glycerol gelatin and captured images. To quantify lipid droplet area, ImageJ software was used. In the cell-based assay, cells were harvested, resuspended, and then seeded onto coverslips inside 6-well plates at a density of 5 × 10^5^ per well. Following cell attachment, the cells underwent a 24-h treatment, after which we performed Oil Red O staining and mounting. The same ImageJ method was used to quantify the lipid droplet area.

#### 2.2.4 Fecal metagenomic analysis

We retrieved frozen stool specimens for subsequent genomic detection. Total microbial genomic DNA was isolated via the E.Z.N.A.® Soil DNA extraction kit. The purity and intactness of extracted nucleic acid were verified through two approaches: OD absorbance quantification on a full-spectrum UV-Vis spectrophotometer, alongside agarose gel electrophoretic separation. DNA passing quality screening underwent mechanical shearing with a Covaris M220 sonicator; target nucleic acid fragments were then purified and recovered. Paired-end (PE) library construction was performed, and after bridge PCR amplification, next-generation sequencing (NGS) was conducted using the Truseq SBS Kit v3-HS reagent. We used the LingEn Biological Cloud Platform to analyze the raw sequencing data (http://www.biozeron.com/), including KEGG functional annotation, assessment of species diversity, inter-group species differences, and generation of related plots.

#### 2.2.5 Untargeted metabolomics assay

Fecal samples were mixed with 950 μL of a methanol-chloroform-ddH_2_O solution (70:20:5, v/v/v), vortexed for 30 s, spiked with internal standards, and vortexed again. The mixed suspension underwent rapid cryogenic quenching with liquid nitrogen, followed by warming to ambient temperature and homogenization via tissue shaking grinding. The homogenate was spun down at 16,000 rpm for 15 min under 4 °C refrigeration; the recovered liquid phase was fully evaporated using vacuum centrifugation. Upon completion of chemical derivatization, another round of centrifugation was carried out, and the clarified upper liquid layer was retained. Both quality control (QC) and test samples were prepared. GC-MS analysis was conducted under constant flow conditions at Shanghai LingEn Biotechnology Co., Ltd., with helium as the carrier gas and an HP-5MS capillary column. A programmed temperature ramp and a mass spectrometry scanning mode were applied for detection. We processed the data using Compound Discoverer 2.1 software, which handled peak extraction, alignment, and other preprocessing steps. After total area normalization, statistical analyses were carried out, including differential metabolite screening and pathway enrichment analysis.

#### 2.2.6 RT-qPCR assay

We homogenized 0.1 g hepatic tissue within 1 mL RNA isolation buffer, supplemented 100 μL chloroform replacement solution and blended thoroughly. The homogenate underwent centrifugation at 12,000 rpm, 4 °C, over a 15-min runtime. Isopropanol was added to the aqueous supernatant to precipitate nucleic acid at −20 °C for 15 min. The isolated RNA precipitate underwent 75% ethanol washing, air dehydration, and final reconstitution in DEPC-treated water. A commercial reverse transcription master mix kit was applied to 1 μg total RNA, with incubation parameters set to 37 °C (15 min) and 85 °C (5 s). Quantitative PCR was performed in 20 μL reaction volumes with the thermal profile: initial 30 s denaturation at 95 °C, then 41 amplification cycles consisting of 5 s at 95 °C and 30 s at 60 °C. GAPDH was selected as the housekeeping gene, and target transcript relative abundances were quantified via the 2^−ΔΔCT^ calculation algorithm. For cellular specimens, culture supernatant t was aspirated, cell monolayers rinsed with PBS, and lysis performed using 1 mL identical RNA isolation buffer; all subsequent manipulations matched the hepatic tissue workflow exactly. The remaining steps were identical to those for liver tissue (Supplementary Tables S1 and S2 for primer sequences of each gene).

#### 2.2.7 Western blot

A total of 0.1 g hepatic and colonic tissue specimens were homogenized in inhibitor-supplemented RIPA lysis buffer containing protease and phosphatase cocktails, followed by 30 min of ice-bath incubation prior to 4 °C centrifugation at 12,000 rpm lasting 5 min. Cell precipitates were processed through an identical workflow, including RIPA lysis, ultrasonic fragmentation and centrifugal separation. Total protein content of each lysate was measured via the BCA colorimetric quantification kit. Specimens were blended with protein loading dye at a 4:1 volume proportion and thermally denatured in a 100 °C water bath for 10 min. Subsequent electrophoretic fractionation was performed on SDS-PAGE gels: an initial 80 V run for 30 min, followed by a 120 V voltage shift for 60 min. Separated protein bands were electrotransferred to PVDF blotting membranes under a steady 300 mA current. Membranes were sealed with 5% skim milk blocking solution over a 2 h incubation period. Then the blots were kept at 4 °C overnight with primary antibody cocktails targeting TLR4 (1:2,000), MyD88 (1:1,000), IκBα (1:5,000), p-IκBα (1:1,000), p65 (1:10,000), p-p65 (1:10,000) and β-actin (1:20,000). After rinsing, blots were incubated with HRP-tagged secondary immunoglobulins diluted 1:5,000 at ambient temperature for 1 h. Target protein luminescence bands were captured with ECL chemiluminescence reagent, and gray-scale values of each band were analyzed with ImageJ software, with β-actin adopted as the internal loading reference.

#### 2.2.8 ELISA

Serum samples were taken from −80 °C storage and allowed to thaw naturally on ice prior to analysis. Hepatic specimens were homogenized with pre-cooled physiological saline at a tissue-solvent mass-volume ratio of 1:9 (g/mL), followed by refrigerated centrifugation at 2,500 rpm and 4 °C over a 20-minute run. The clarified upper liquid layer was retained for subsequent biomarker detection. For cellular cultures, cell culture medium was gathered and subjected to 3,000 rpm centrifugation at 4 °C for 20 min. We determined the abundance of LPS, IL-1β, IL-6 and TNF-α within serum, hepatic homogenate and cell culture media via ready-to-use biochemical assay kits. All experimental operations, including sample loading, incubation, plate washing, chromogen addition, reaction termination and absorbance measurement, strictly complied with the official operational guidelines supplied by each reagent supplier.

### 2.3 Statistical evaluation

All measured experimental datasets were analyzed via SPSS Statistics 27.0 software. Measurement outcomes were expressed as mean ± standard deviation $\left(\bar{x}\pm \;S\right)$ . One-way ANOVA was adopted for multi-group comparisons when datasets conformed to normal distribution and homogeneity of variance, with Fisher’s least significant difference (LSD) *post hoc* test applied for subsequent pairwise intergroup contrasts following a significant ANOVA. Non-parametric Kruskal–Wallis H test was utilized to evaluate intergroup disparities for data deviating from Gaussian distribution. Any difference yielding *P* < 0.05 was defined as statistically significant. All figures were generated using GraphPad Prism 9.5.

## 3 Results

### 3.1 Effects of DOP on liver histopathology and cellular lipid deposition in MAFLD rats

After the 12-week modeling period (Figure 1A), hepatic tissue collected from control group C displayed intact histological architecture, lacking fatty degeneration and inflammatory cellular infiltration. By comparison, model group M presented widespread hepatocellular steatosis, cellular ballooning injury and massive lipid vacuole deposition (*P* < 0.001), which verified the stable construction of the MAFLD animal phenotype. Relative to untreated model rats, supplementation with DOP significantly lowered whole-body mass, hepatic weight and liver coefficient in MAFLD animals. The compound also relieved hepatic pathological damage (Supplementary Figure S1) and mitigated excessive lipid droplet buildup within liver parenchyma (*P* < 0.05, *P* < 0.01, *P* < 0.001).

**FIGURE 1:**
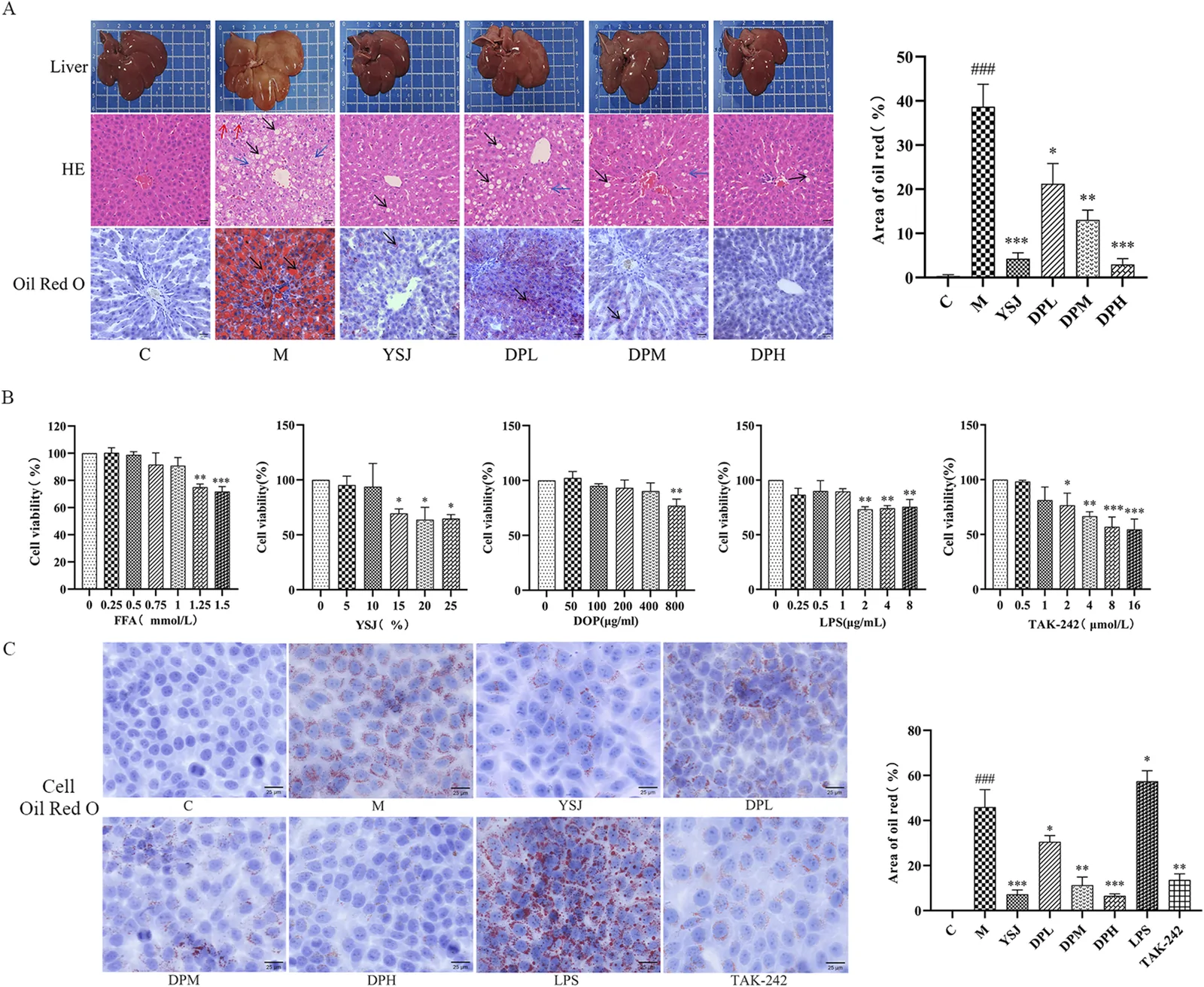
Effects of DOP on hepatic pathology and lipid accumulation in MAFLD rats and hepatocytes. **(A)** Effects of DOP on liver morphology and histopathological changes in MAFLD rats (×400, scale bar = 25 μm). **(B)** Effects of various treatments on HepG2 cell viability ( $\bar{x}\pm S$ , n = 3). **(C)** Oil Red O staining of cells (×400, scale bar = 25 μm). Notes: Black arrows point to large fat vacuoles (HE staining) as well as red lipid droplets (Oil Red O staining); blue arrows denote small lipid droplets; red arrows indicate mild ballooning degeneration. Statistical significance: vs. C group, ^#^ *P* < 0.05, ^##^ *P* < 0.01, ^###^ *P* < 0.001; vs. M group, ^*^ *P* < 0.05, ^**^ *P* < 0.01, ^***^ *P* < 0.001. The same notations apply to subsequent figures.

Cell viability tests showed that FFA levels above 1 mmol/L caused a significant drop in viability (*P* < 0.01); therefore, 1 mmol/L was selected for establishing the MAFLD cell model. Based on the survival rates across groups, the following concentrations were adopted for subsequent experiments: 10% for YSJ cell-free extract, 100, 200, and 400 μg/mL for low-, medium-, and high-dose DOP, respectively, 1 μg/mL for LPS, and 1 μmol/L for TAK-242 (Figure 1B).

Oil Red O staining (Figure 1C) showed that, relative to group C, both group M and the LPS-treated group displayed extensive intracellular lipid droplet accumulation (*P* < 0.001). Compared with group M, each DOP dose group and the TAK-242 group exhibited significantly fewer intracellular lipid droplets and less lipid deposition (*P* < 0.05, *P* < 0.01, *P* < 0.001). Quantitative analysis of stained areas further demonstrated that the LPS group possessed significantly broader lipid droplet coverage compared with group M (*P* < 0.05), which demonstrates that LPS exacerbates intracellular lipid deposition.

### 3.2 Effects of DOP on serum lipids, liver function, hepatic lipids, and hepatocellular injury in MAFLD rats

Relative to M Group, intervention with DOP markedly downregulated TC, TG and LDL-C concentrations within serum, hepatic tissue and cultured hepatocytes (*P* < 0.05, *P* < 0.01, *P* < 0.001). Meanwhile, HDL-C contents were elevated in serum and liver samples with distinct statistical differences (*P* < 0.05, *P* < 0.001) as illustrated in Figures 2A–D. For cellular specimens, HDL-C concentrations presented a mild rising tendency without attaining statistical significance (*P* > 0.05). With respect to hepatic function indexes and cellular damage markers, DOP supplementation produced dose-dependent declines in ALT and AST concentrations across serum, liver tissue and cell lysates, alongside reduced serum GGT values (*P* < 0.05, *P* < 0.01, *P* < 0.001). Of all DOP dosing subgroups, the high-dose DPH cohort displayed the strongest therapeutic ameliorative effect.

**FIGURE 2:**
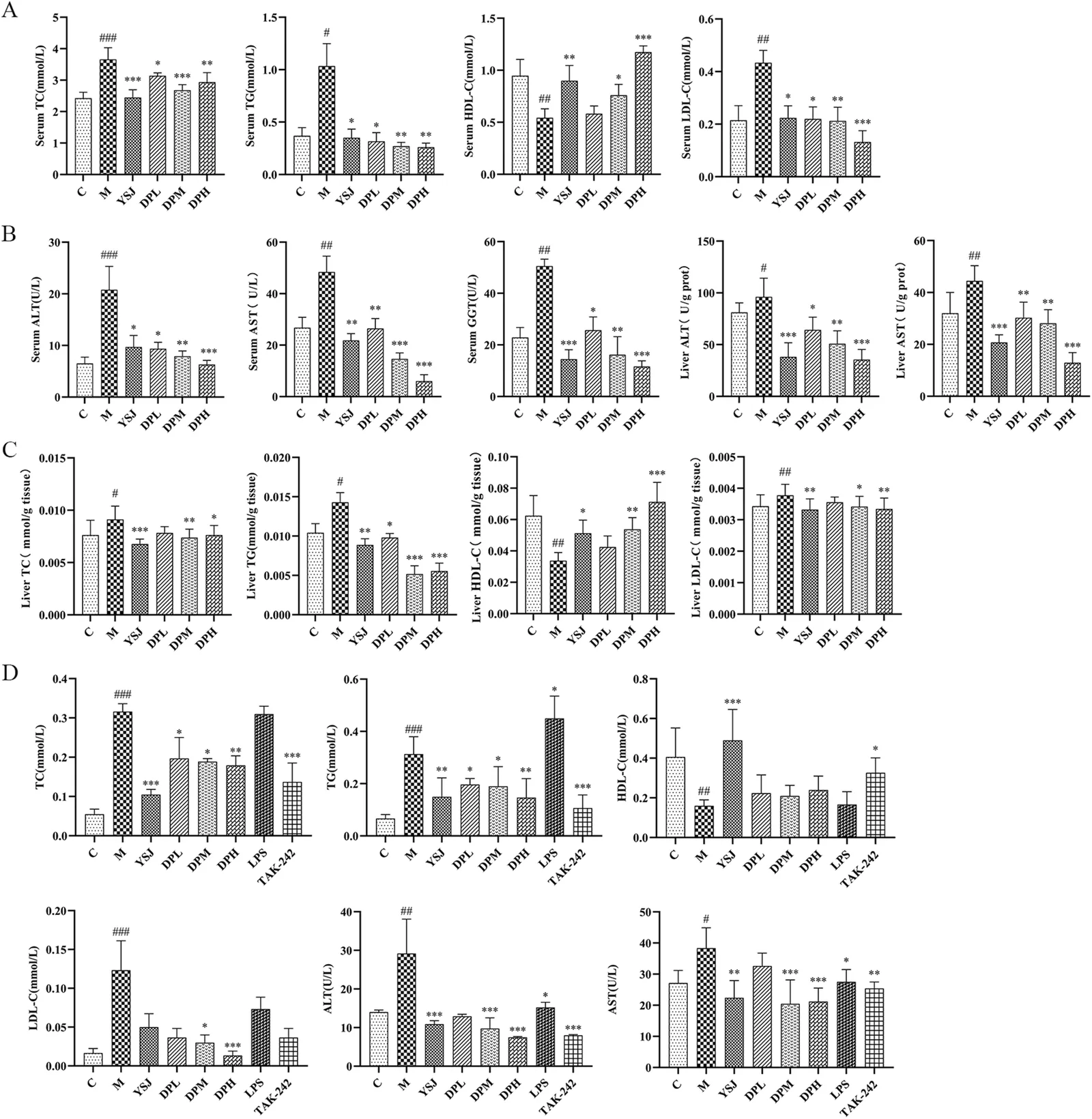
Regulatory impacts of DOP upon serum lipid profiles, hepatic function indices, intrahepatic lipid deposition and hepatocellular damage within MAFLD rats models and *in vitro* cultured hepatocytes. **(A)** Serum lipid levels in different groups of rats. **(B)** Liver function indicators in each rat group. **(C)** Hepatic lipid content among experimental rat groups ( $\bar{x}\pm S$ , n = 8). **(D)** Levels of biochemical markers in cells under various treatments ( $\bar{x}\pm S$ , n = 3).

### 3.3 Modulatory impacts of DOP on intestinal microbial composition in MAFLD rats models

DOP effectively ameliorated the structural imbalance of intestinal microbiota in HFD-induced MAFLD rats, restored the richness and diversity of gut microbes, and stabilized the community structure of intestinal microecology (Supplementary Figure S2). High-throughput metagenomic sequencing detected 146 distinct phyla, 5,666 genera and 24,646 microbial species in total. After filtering out unannotated taxonomic units, 3,398 annotated species were retained for subsequent statistical analysis. All taxa holding less than 1% relative abundance were combined under the collective category labeled “Other”. In contrast to untreated model rats (Group M), DOP supplementation sharply lowered the relative proportions of three harmful phyla: Bacillota (previously named *Firmicutes*), *Desulfobacterota* and Gram-negative *Pseudomonadota* (*P* < 0.05, *P* < 0.01, *P* < 0.001). Correspondingly, the F/B ratio also declined remarkably following DOP administration (*P* < 0.05) (Figures 3A–E). At the genus taxonomic rank, DOP supplementation suppressed the proliferation of multiple opportunistic pathogenic microbes, such as *Parasutterella*, *Marinomonas*, *Paramuribaculum*, *Allobaculum*, *Bulleidia* and *Duncaniella*. Meanwhile, the compound replenished the population of butyrate-generating commensal taxa including *Coprococcus*, *Agathobacter* and *Butyribacter* (*P* < 0.05, *P* < 0.01, *P* < 0.001) (Figures 4A–E). At the species level, DOP downregulated the Gram-negative bacterium *Brachyspira aalborgi* and the harmful species *Acetobacterium fimetarium* (*P* < 0.05), while significantly enriching beneficial probiotics including *Dysosmobacter welbionis*, *Ligilactobacillus animalis*, and *Limosilactobacillus balticus* (*P* < 0.05, *P* < 0.01) (Figures 5A–C).

**FIGURE 3:**
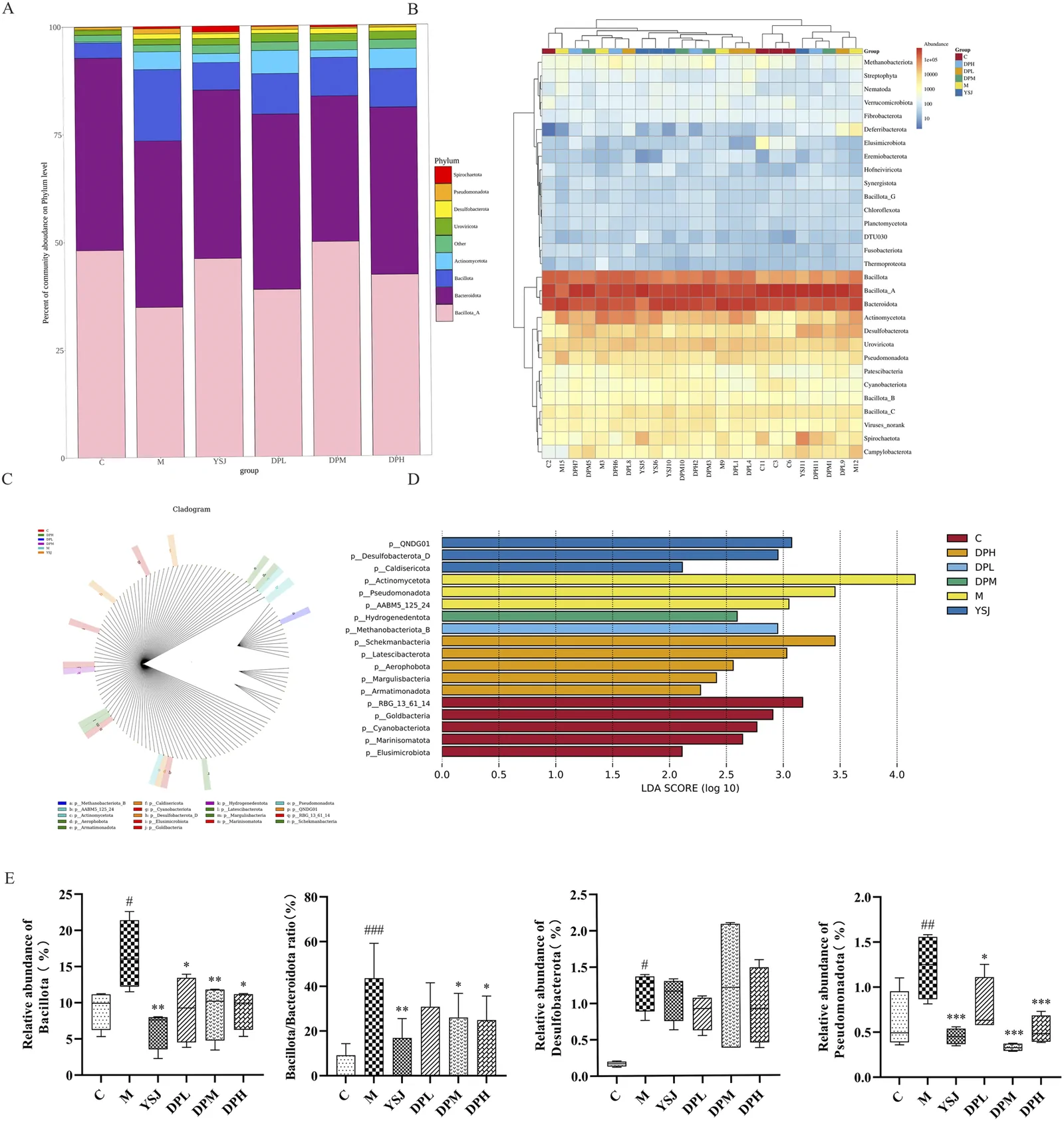
Phyla-level composition and differential analysis of the intestinal microbial communities in all experimental cohorts ( $\bar{x}\pm S$ , n = 4). **(A)** Abundance of bacterial phyla. **(B)** Correlation heatmap at the phyla level. **(C)** Cladogram showing phyla-level biomarkers. **(D)** LEfSe-based discrimination of phyla-level differences. **(E)** Analysis of differentially abundant phyla among groups.

**FIGURE 4:**
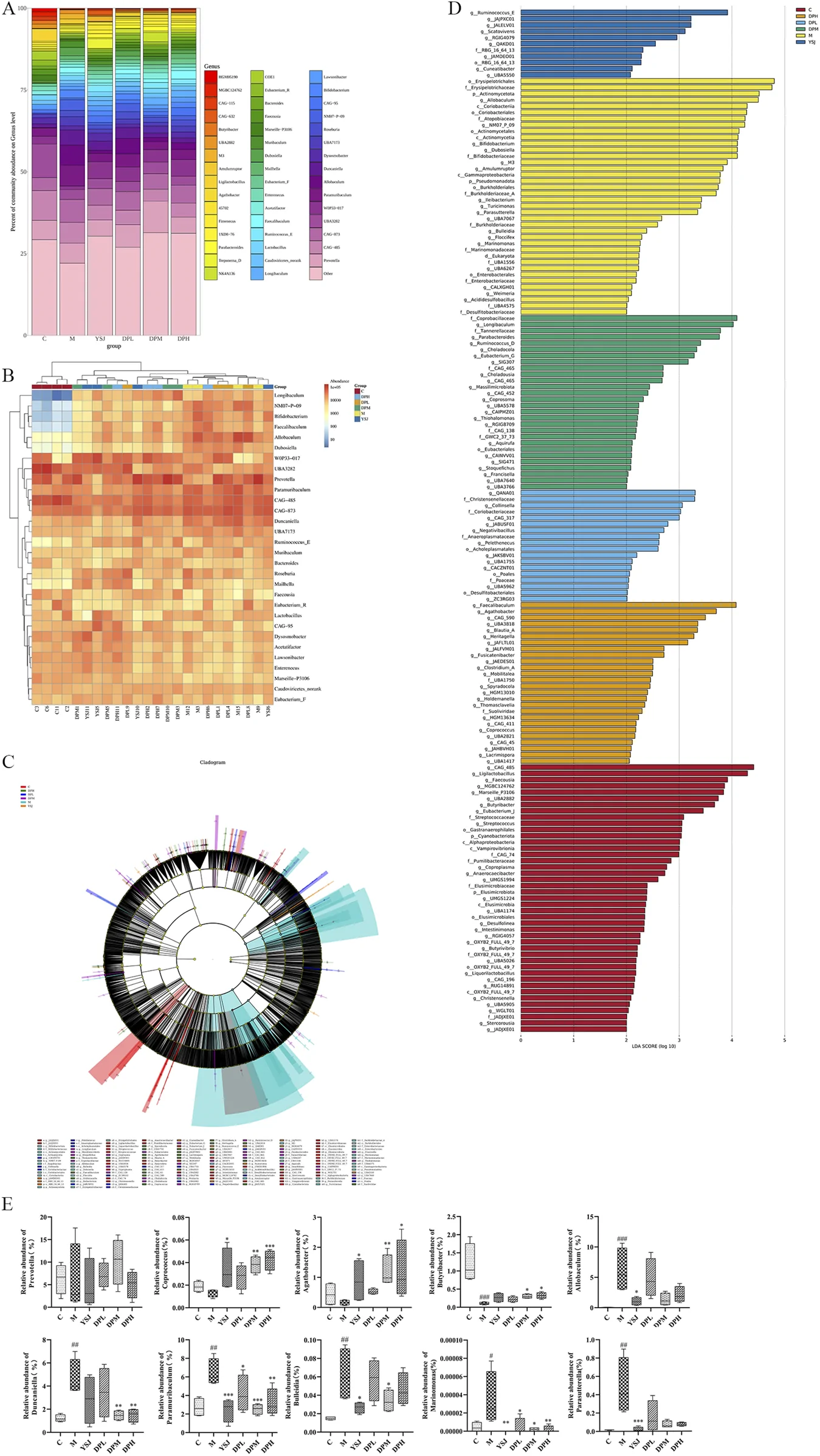
Composition and intergroup variance comparison of intestinal bacterial communities Genus-rank across experimental groups ( $\bar{x}\pm S$ , n = 4). **(A)** Genus-level community abundance. **(B)** Heatmap of genus-level correlations. **(C)** Cladogram showing genus-level biomarkers identified by LEfSe. **(D)** LEfSe-based differential analysis of bacterial genera. **(E)** Analysis of differentially abundant genera among groups.

**FIGURE 5:**
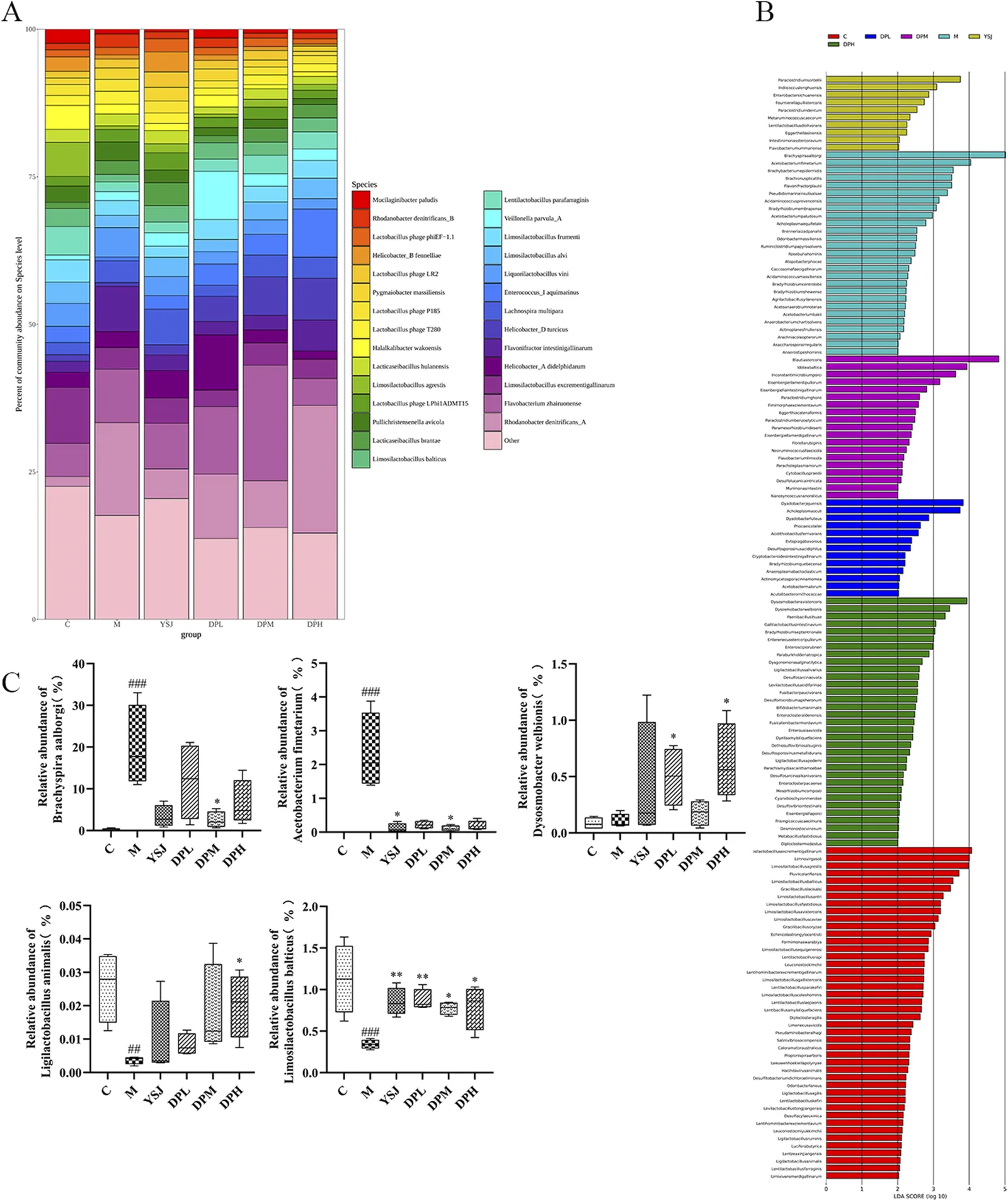
Species-level gut microbiota composition and differential analysis among rat groups ( $\bar{x}\pm S$ , n = 4). **(A)** Community abundance of bacterial species. **(B)** LEfSe analysis for species-level differences. **(C)** Differentially abundant species.

### 3.4 COG functional and KEGG pathway enrichment analyses

Figure 6A presents the COG functional abundance analysis, showing marked differences in functional enrichment when comparing the intestinal microbial communities of group C and group M. Both groups exhibited high abundances of genes involved in “function unknown,” “replication,” and “signal transduction mechanisms.” Group M showed a significantly lower abundance in the “defense mechanisms” category Relative to healthy control rats (*P* < 0.05). KEGG pathway analysis (Figure 6B) showed that metabolic pathways dominated the functional profiles across all samples. Further examination (Figures 6C,D) indicated that at Level 2, Lipid- and amino-acid-related metabolic pathways showed predominant enrichment. At Level 3, “metabolic pathways” displayed the highest abundance, while the glycolysis/gluconeogenesis pathway was also broadly enriched across samples.

**FIGURE 6:**
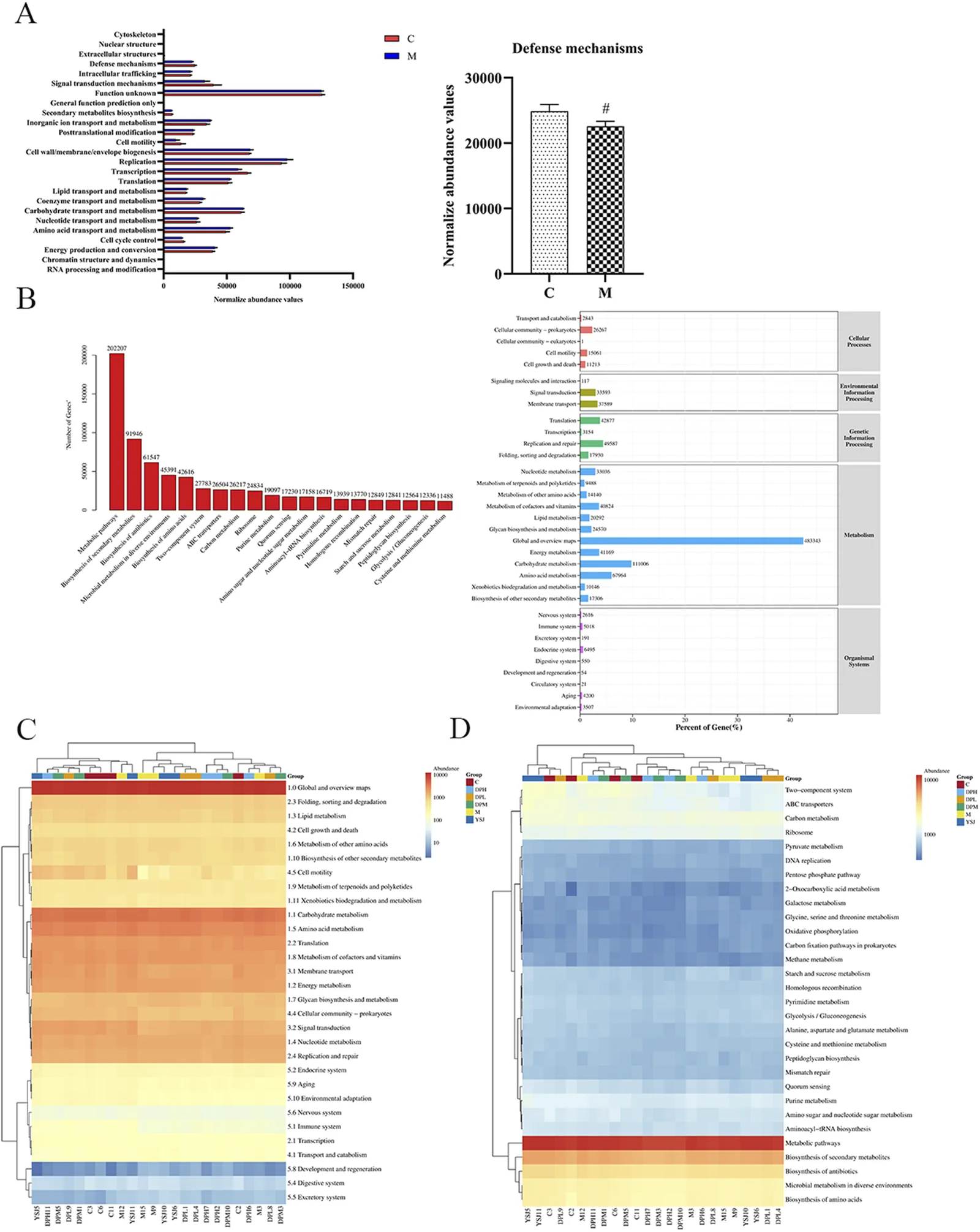
Enrichment analysis of COG functions and KEGG pathways ( $\bar{x}\pm S$ , n = 4). **(A)** Annotated COG functional enrichment profile for group C versus group M. **(B)** Overview of KEGG pathway annotations. **(C)** Heatmap of KEGG pathway abundance at Level-2 categories. **(D)** Heatmap of KEGG pathway abundance at Level-3 categories.

### 3.5 Effects of DOP regarding gut metabolites in MAFLD rats

PCA revealed a trend of within-group clustering and between-group separation among the experimental groups (Figure 7A). The C and M groups were clearly separated, and all treatment groups shifted toward the C group. Volcano plots and differential metabolite statistics (Figure 7B) showed that, compared with group C, group M exhibited 823 upregulated and 326 downregulated metabolites. Using group M as a reference, the YSJ, DPL, DPM, and DPH groups yielded 277, 93, 342, and 253 differential metabolites, respectively. Notably, ASP-ASP levels were decreased in group M but were significantly restored after DPL and DPH treatment.

**FIGURE 7:**
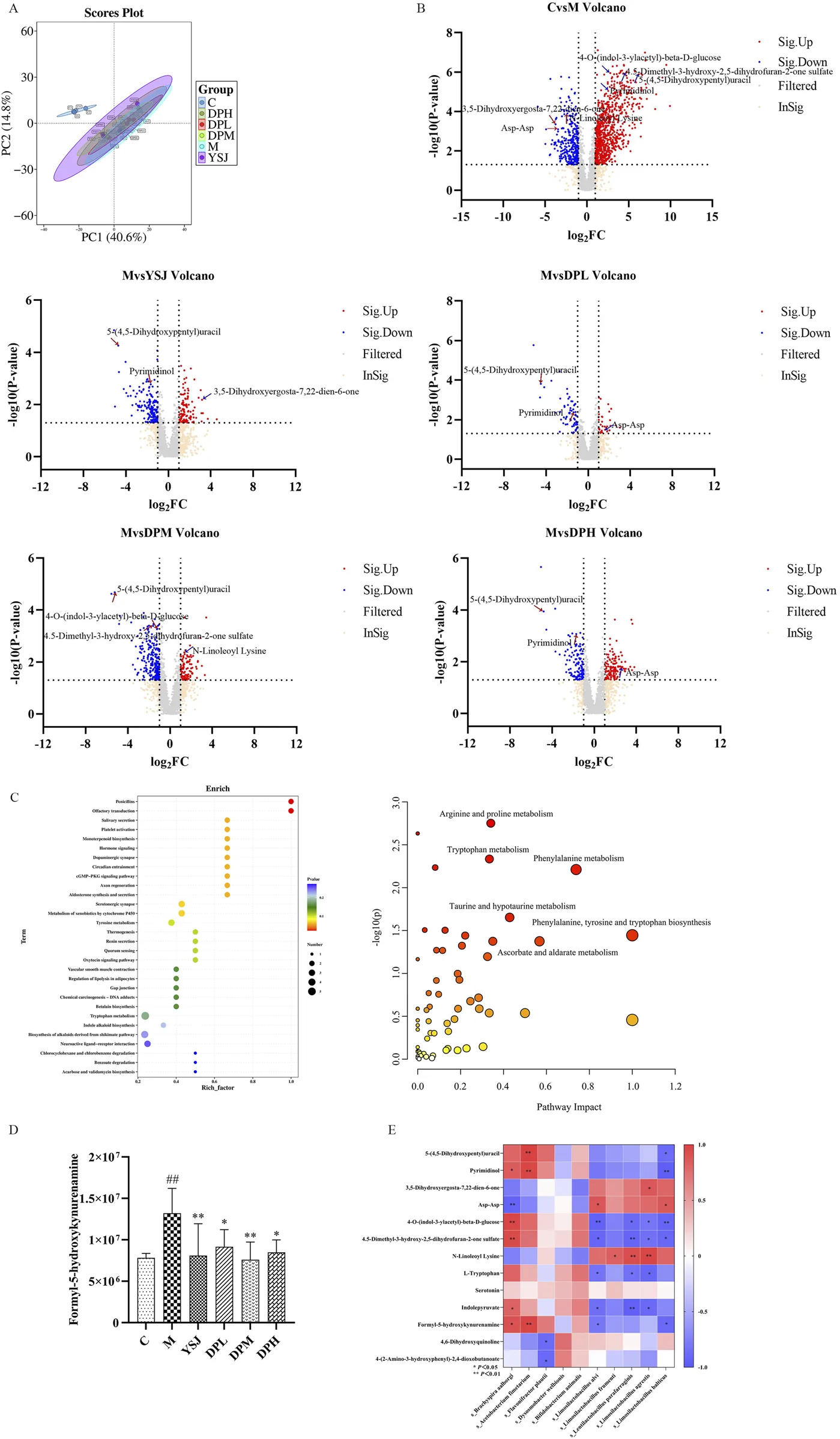
Effects of DOP on distinct metabolites in MAFLD rats ( $\bar{x}\pm S$ , n = 4). **(A)** PCA score scatter plots of all groups. **(B)** Volcano plots showing differential metabolites across groups. **(C)** Pathway analysis of metabolites differing between groups. **(D)** Abundance of differential metabolite formyl-5-hydroxykynurenamine. **(E)** Correlation analysis at the species level between differentially abundant bacteria and differential metabolites.

KEGG functional enrichment profiling identified tryptophan catabolism as a conserved central metabolic cascade across all experimental cohorts. Among its metabolites, formyl-5-hydroxykynurenamine was exhibited prominent accumulation in group M (*P* < 0.01) and significantly decreased DOP supplementation (*P* < 0.05, *P* < 0.01) (Figures 7C,D). Further pathway enrichment analysis (MetaboAnalyst 6.0) with screening criteria set to VIP > 1 and *P* < 0.05 showed that DOP administration exerted regulatory impacts on multiple amino acid-related metabolic cascades arginine-proline turnover, tryptophan catabolism, phenylalanine breakdown, the biosynthetic route for phenylalanine-tyrosine-tryptophan, alongside taurine-hypotaurine biotransformation pathways.

Pearson correlation analysis (Figure 7E) revealed that ASP-ASP was significantly negatively correlated with the harmful bacterium *B. aalborgi* (*P* < 0.001) and positively correlated with the beneficial bacteria *L. alvi* and *L. balticus* (*P* < 0.05). L-Tryptophan displayed prominent inverse associations with the probiotics *L. alvi*, *L. parafarraginis*, and *L. agrestis* (*P* < 0.05). Additionally, formyl-5-hydroxykynurenamine was presented strong positive covariation with the harmful bacteria *Brachyspira aalborgi* and *Acetobacterium fimetarium* (*P* < 0.05, *P* < 0.01), and negatively associations with *L. alvi* and *L. balticus* (*P* < 0.05).

### 3.6 DOP improves intestinal barrier function in MAFLD rats

Intestinal barrier integrity serves as a critical checkpoint of the microbiota-gut-liver axis, and impaired intestinal permeability is widely recognized as an important driver for the progression of MAFLD (Liu et al., 2023; Cui et al., 2025). Compared with untreated model rats, DOP supplementation substantially elevated the colonic protein abundance of tight junction proteins Claudin-1 and Occludin. Quantitative analysis revealed obvious -fold upregulation of Claudin-1 and Occludin in DOP-treated groups, accompanied by significant statistical differences (*P* < 0.01, *P* < 0.001), By contrast, circulating DAO and D-LA contents displayed prominent descending trends with statistical differences (*P* < 0.05, *P* < 0.01, *P* < 0.001) (Figure 8A). In combination with preceding multi-omics data, these intestinal barrier benefits are likely linked to DOP-mediated remodeling of gut microbiota and shifts in intestinal metabolites, which further restrain excessive translocation of microbial-derived stimuli and alleviate liver inflammatory injury (Fernandez-Cantos et al., 2021; Chen and Ho, 2023).

**FIGURE 8:**
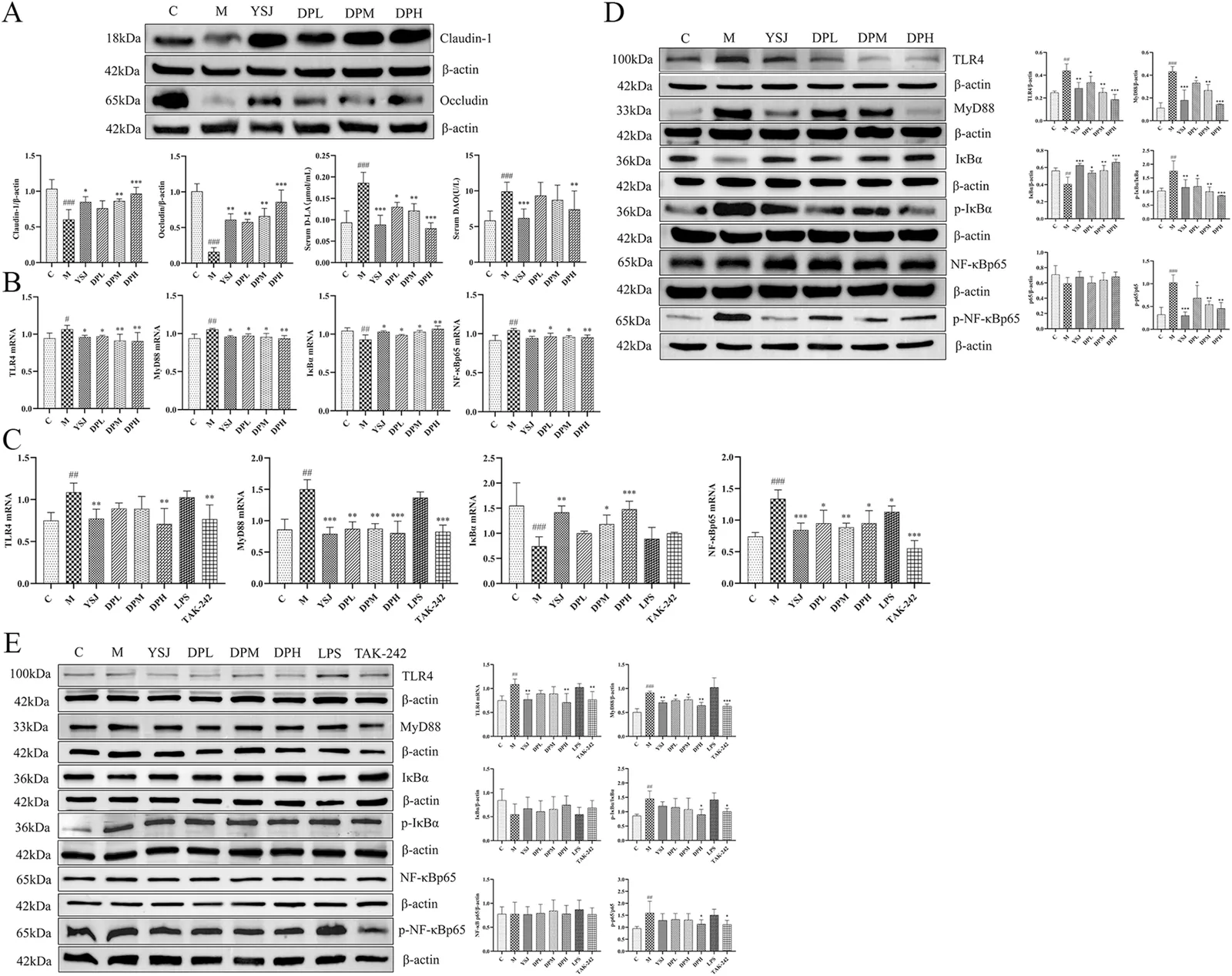
Effects of DOP on intestinal permeability and the TLR4/NF-κB pathway in a rat model of MAFLD. **(A)** Assessment of intestinal permeability across rat groups ( $\bar{x}\pm S$ , n = 3). **(B)** Relative gene expression levels in rat tissues ( $\bar{x}\pm S$ , n = 8). **(C)** Gene expression profiles in cultured cells from each group ( $\bar{x}\pm S$ , n = 3). **(D)** Protein expression levels in rat samples ( $\bar{x}\pm S$ , n = 3). **(E)** Protein abundance in cells derived from different experimental groups ( $\bar{x}\pm S$ , n = 3).

### 3.7 DOP modulates transcript and protein abundance of TLR4/NF-κB signaling molecules in hepatic tissue and hepatocytes from MAFLD model rats

At the transcriptional level (Figures 8B,C), DOP administration in MAFLD rats led to reduced mRNA abundance of TLR4, MyD88, and NF-κB p65 within both hepatic tissue and cultured hepatocytes, accompanied by a marked rise in IκBα mRNA (*P* < 0.05, *P* < 0.01, *P* < 0.001). Western blot quantification (Figures 8D,E), DOP intervention lowered TLR4 and MyD88 protein amounts, alongside the phosphorylation ratios of IκBα and p65, to varying extents in both hepatic tissue and cultured hepatocytes (*P* < 0.05, *P* < 0.01, *P* < 0.001). Total IκBα protein was significantly recovered in liver tissue (*P* < 0.05, *P* < 0.01, *P* < 0.001) and showed a rising trend in cells, yet this shift failed to reach statistical threshold (*P* > 0.05). These effects were dose-dependent, with the DPH group producing the greatest response.

### 3.8 DOP alleviates pro-inflammatory mediator levels in serum, liver, and cells of MAFLD rats

Relative to untreated model rats, DOP administration drastically lowered the contents of LPS, IL-1β, IL-6, and TNF-α across three sample matrices: circulating serum, hepatic tissue homogenates and cellular culture supernatants (*P* < 0.05, *P* < 0.01, *P* < 0.001) (Figures 9A–C).

**FIGURE 9:**
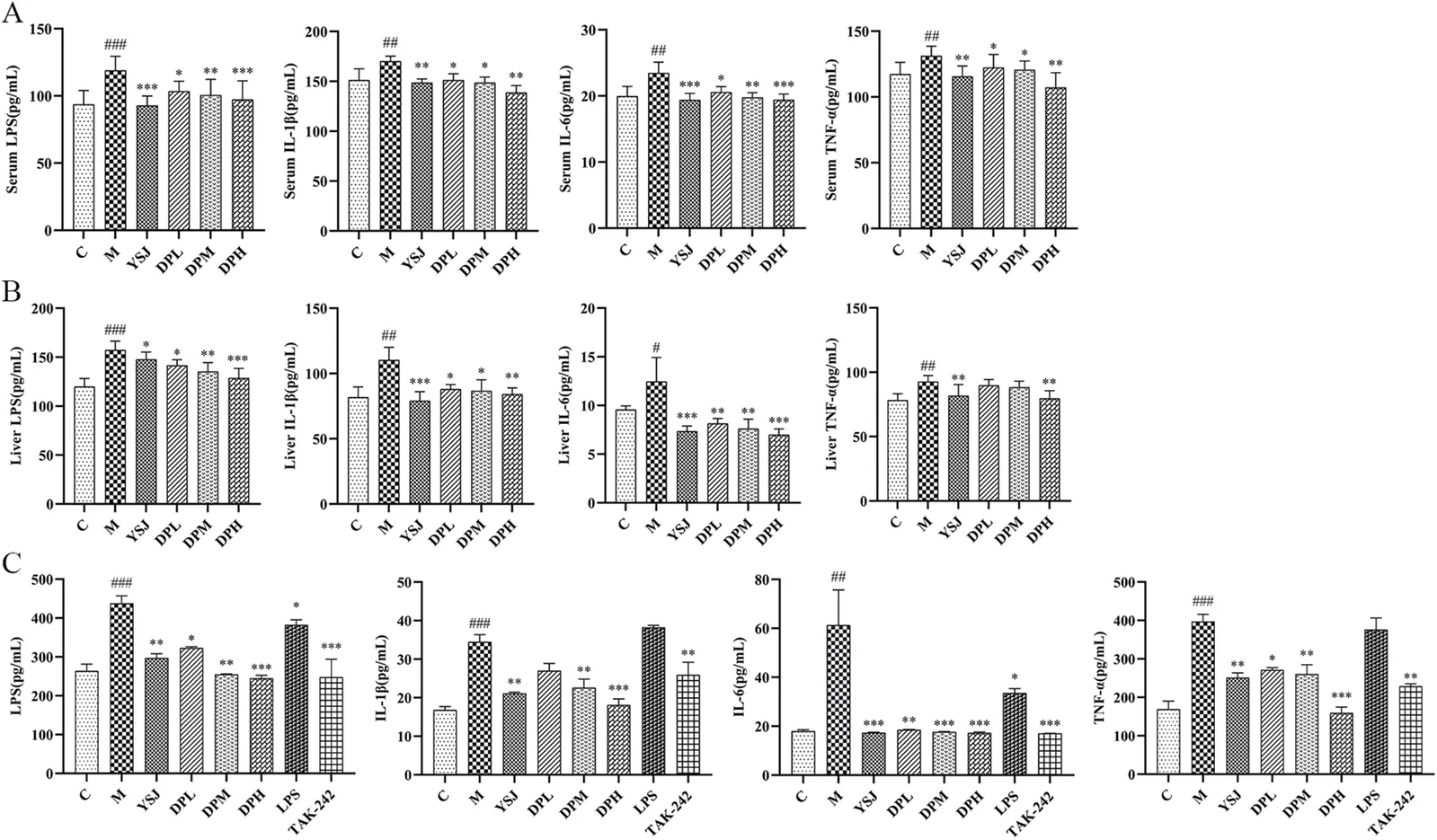
Effects of DOP on inflammatory cytokines in serum, liver, and cells of MAFLD rats. **(A)** Inflammatory cytokine levels quantified in serum for all groups. **(B)** Corresponding cytokine concentrations detected in liver tissues. **(C)** Cytokine abundance determined in cell culture samples.

### 3.9 Correlation analysis among gut microbiota, metabolites, and measured indicators

Pearson correlation analysis (Figure 10A) indicated that certain Gram-negative bacteria—*Pseudomonadota*, *Marinomonas*, *Parasutterella*, and *B. aalborgi* exhibited strong positive associations with serum LPS, IL-6, D-LA, and hepatic TLR4 (*P* < 0.05, *P* < 0.01). On the contrary, *Bacillota* and *Parasutterella* marked inverse relationships with the intestinal barrier structural proteins Claudin-1 and Occludin (*P* < 0.05, *P* < 0.01). The probiotic taxa *L. alvi* and *L. balticus* were strongly inversely associated with circulating LPS and D-LA biomarkers, whereas they correlated positively with Claudin-1 and Occludin (*P* < 0.05, *P* < 0.01).

**FIGURE 10:**
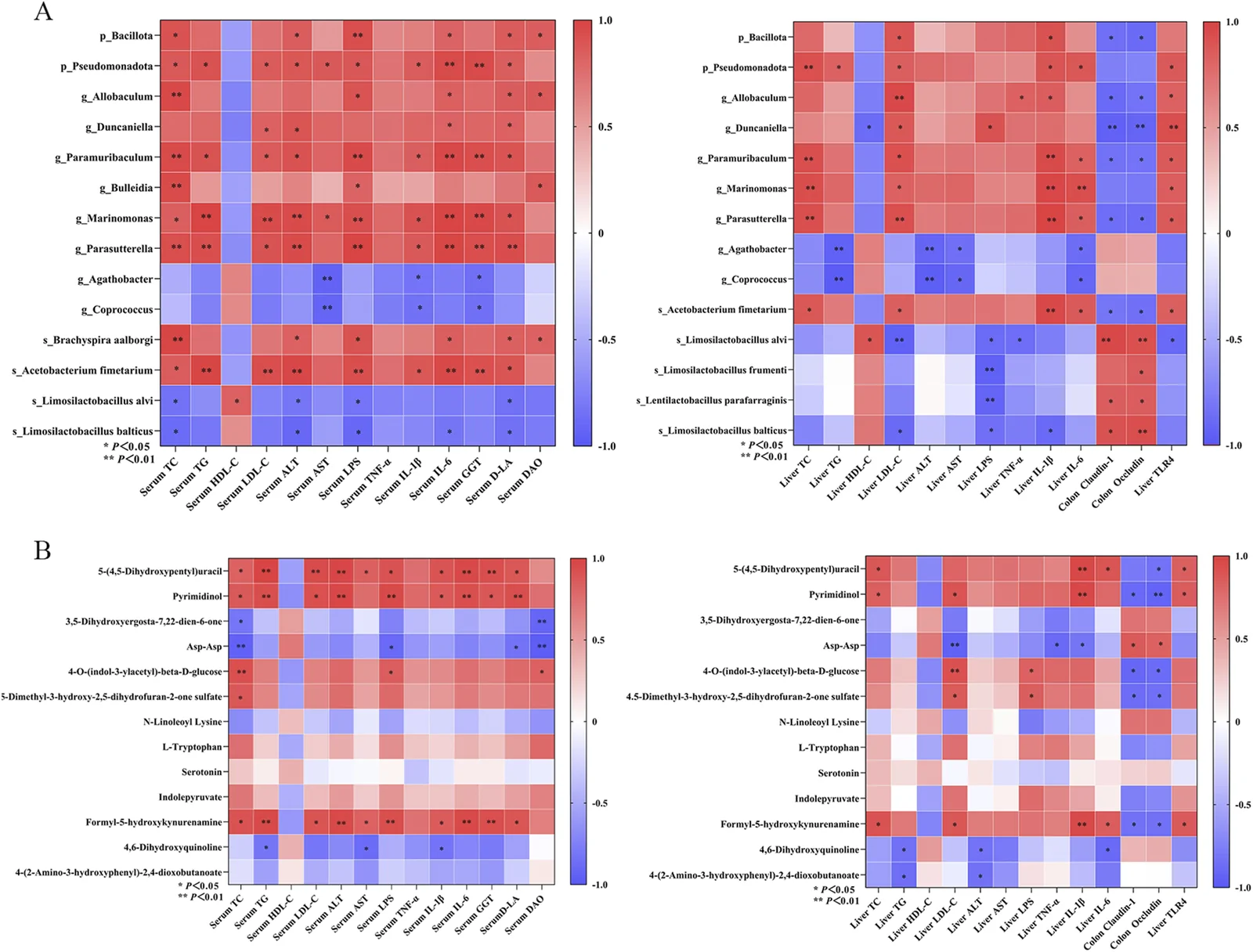
Interrelations among gut flora, metabolites, and physiological parameters in animals. **(A)** Correlations between animal physiological indices and across three taxonomic tiers: phylum, genus and species. **(B)** Associations of differential metabolites with the same animal parameters.

Regarding metabolites (Figure 10B), Formyl-5-hydroxykynurenamine, a tryptophan metabolite that was upregulated in the M group, was strongly positively associated with circulating LPS, D-LA and hepatic TLR4 (*P* < 0.05), while being inversely linked to the intestinal barrier proteins Claudin-1 and Occludin (*P* < 0.05). In contrast, the downregulated metabolite ASP-ASP in the M group correlated negatively with the above inflammatory markers and positively with gut tight junction structural proteins (*P* < 0.05, *P* < 0.01).

## 4 Discussion

According to recent pharmacological studies, DO possesses multiple biological activities, such as liver protection, anti-oxidation, anti-inflammation and glycolipid regulation (Zhou R. et al., 2025; Liu et al., 2025). In recent years, plant polysaccharides have attracted widespread attention as promising natural agents for the prevention and treatment of liver diseases (Wei et al., 2024; Zhang et al., 2025). Existing research revealed that carbohydrate polymers extracted from *Dendrobium officinale* are capable of reshaping gut microbiota, elevating SCFAs production and suppressing NF-κB-dependent inflammatory responses (Shi et al., 2025). Our previous research has verified that DO can relieve MASH accompanied by synchronous improvements in gut flora structure, intestinal barrier integrity and the TLR4/NF-κB signaling pathway. As a water-soluble macromolecular polysaccharide, DOP does not directly penetrate the hepatocyte membrane; instead, it exerts its pharmacological effects through the gut-liver axis (Wan et al., 2025). Concurrently, DOP reinforces intestinal barrier integrity by upregulating tight junction proteins (ZO-1, occludin, claudin-1), thereby reducing lipopolysaccharide (LPS) translocation and subsequent TLR4/NF-κB activation in the liver (Tao et al., 2025; Bian et al., 2026). Additionally, DOP modulates gut microbiota composition and tryptophan metabolism, further contributing to the attenuation of hepatic inflammatory and metabolic disturbances via the AhR signaling pathway.

Accordingly, the present study focused on gut microbiota and intestinal metabolite profiles to explore the protective effects of DOP against MAFLD in SD rats based on gut-liver axis theory. The pathogenesis of MAFLD is widely explained by the “multiple-hit theory,” in which intestinal dysbiosis serves as a critical predisposing factor (Clare et al., 2022). Growing evidence indicates that herbal extracts exert protective effects against MAFLD by remodeling gut microbiota and further inhibiting the TLR4/NF-κB signaling axis (Lv C. et al., 2024). Intestinal microbial imbalance increases gut permeability, allowing LPS and other hepatotoxic substances to enter the liver through portal circulation. Hepatic LPS accumulation triggers TLR4/MyD88 signaling activation, induces IκBα degradation and NF-κB p65 nuclear translocation, and ultimately promotes the secretion of pro-inflammatory cytokines, thereby driving the progression of hepatic steatosis and inflammation (Tam et al., 2021; Wang et al., 2020; Chen et al., 2024; Ferro et al., 2020; Ohtani et al., 2023). This series of events ultimately promotes the onset and aggravation of MAFLD. Therefore, inhibiting excessive activation of the TLR4/NF-κB pathway has become a potential strategy to alleviate MAFLD-related liver damage. LPS enriched on the outer membrane of Gram-negative bacteria can directly activate hepatic TLR4/NF-κB signaling and induce inflammatory responses (Gu et al., 2023). As the primary energy source for colon epithelial cells, butyrate maintains the integrity of tight junctions and intestinal immune homeostasis (Du et al., 2025; Rong and Shu, 2024; Lv X. et al., 2024; Mann et al., 2024).

Through metagenomic analysis of the gut microbiota, our research team observed that DOP administration could restore gut diversity in MAFLD rats. Specifically, it reduced the F/B ratio and decreased the relative abundance of harmful Gram-negative bacteria, including *Pseudomonadota*, *Parasutterella*, and *B. aalborgi*. Meanwhile, we observed a rise in the abundance of probiotics that produce short-chain fatty acids, such as the genera *Butyribacter* and *Agathobacter*, as well as the species *D. welbionis* and *L. animalis*. Further functional annotation using the COG database revealed that the “defense mechanisms” functional category exhibited a significantly lower abundance in the MAFLD model group. KEGG functional enrichment analysis, the functions of the intestinal flora were predominantly associated with metabolic cascades. At Level 2, the enriched signatures largely clustered around amino acid turnover, lipid catabolism and associated biochemical reactions. Notably, amino acid metabolites—such as amines, indoles, and sulfur-containing compounds—act as vital inter-organ signaling mediators across the gut-liver crosstalk axis and can influence the progression of liver diseases by alter host immune homeostasis and metabolic status (Luo et al., 2025; Hu et al., 2025). In addition, abnormal lipid metabolites can act as endogenous TLR4 ligands to trigger hepatic inflammatory responses (Ren et al., 2024; Patel et al., 2026). On this basis, dysbiosis-driven disorders of lipid metabolism may further enhance the production and accumulation of the aforementioned TLR4 ligands, consequently promoting the malignant transformation from MAFLD to MASH (Bahitham et al., 2025). The present microbial sequencing results suggested that DOP-mediated microbial improvement was closely correlated with reduced LPS-related inflammatory responses, although causal verification remains insufficient.

We further analyzed differential intestinal metabolites across each group. The results showed DOP intervention significantly upregulated ASP-ASP and downregulated formyl-5-hydroxykynurenamine, a key metabolite of the tryptophan-kynurenine pathway. KEGG enrichment identified tryptophan metabolism as the core metabolic pathway regulated by DOP, with the kynurenine branch as its main downstream route. Correlation analysis uncovered significant statistical associations between gut microbes and target metabolites. ASP-ASP was negatively correlated with the harmful Gram-negative strain *B. aalborgi*, and a positive correlation with *Lactobacillus*, while formyl-5-hydroxykynurenamine showed the opposite correlation trend. Existing studies have confirmed that tryptophan metabolism acts as a critical signaling bridge connecting gut microbiota and host immunity, and its imbalance accelerates the progression from MAFLD to MASH (Chen et al., 2025; Luo et al., 2026). Inflammatory stimulation increases IDO activity and shifts tryptophan metabolism toward the kynurenine branch, which impairs intestinal barrier function and aggravates hepatic steatohepatitis (Tan et al., 2025; Teunis et al., 2022; Taleb, 2019). Notably, kynurenine metabolites themselves are bioactive signaling molecules that activate aryl hydrocarbon receptor (AhR) and promote anti-inflammatory responses (Grishanova and Perepechaeva, 2024), with emerging crosstalk to TLR4/NF-κB signaling (Ala et al., 2022). Likewise, aspartate–the monomer of ASP-ASP–has been shown to improve intestinal barrier and inhibit TLR4/NF-κB activation (Wang et al., 2017). These findings support the observed correlations, but direct evidence for ASP-ASP and formyl-5-hydroxykynurenamine as upstream regulators of the gut-liver axis requires further functional validation, including metabolite supplementation and rescue experiments.

Intestinal barrier dysfunction and increased gut permeability are key initiating events in MAFLD progression and are closely related to hepatic inflammatory severity (Gu et al., 2023; Du et al., 2025). We evaluated intestinal barrier integrity to assess gut permeability changes, MAFLD rats given DOP showed elevated colonic abundance of two core barrier structural proteins: Claudin-1 and Occludin, two core barrier structural proteins. In addition, serum levels of DAO and D-LA were reduced, and leakage of hepatotoxic substances such as LPS was attenuated. Pearson correlation analysis further revealed that gut-derived LPS may predominantly originate from Gram-negative pathogenic bacteria, including *Pseudomonadota*, *Marinomonas*, *Parasutterella*, and *B. aalborgi*. Notably, the metabolite Formyl-5-hydroxykynurenamine was positively linked to serum LPS, D-LA, and hepatic TLR4 protein to a significant degree, while showing a significant negative correlation with colonic Claudin-1 and Occludin proteins. Taken together, all correlative data show that DOP intervention is accompanied by improved intestinal flora, normalized metabolite levels, recovered intestinal barrier and reduced LPS leakage. Such synchronous variations provide correlative evidence linking gut homeostasis improvement to DOP’s liver-protective phenotypes, while causal proof needs further functional experiments.

LPS functions as a key initiator of the TLR4/NF-κB signal transduction, acting as a core molecular bridge linking intestinal inflammation with liver injury (Ren et al., 2024; Zhou M. et al., 2025). When this pathway is suppressed, marked amelioration of MAFLD can be achieved (Ma et al., 2026; Alam et al., 2025). In this study, DOP treatment significantly downregulated the transcriptional and translational expression of TLR4, MyD88, and NF-κB p65, while elevating IκBα levels and reducing serum and hepatic LPS accumulation, accompanied by decreased pro-inflammatory factor release. Multiple natural phytochemicals have been proven to ameliorate HFD-induced hepatic lipid accumulation via suppressing the TLR4/MyD88/NF-κB inflammatory cascade (Zhou X. et al., 2025). Consistent with *in vivo* findings, *in vitro* cell experiments further verified that DOP alleviated hepatocyte steatosis and inflammatory responses via inhibiting TLR4/NF-κB pathway activation.

Multiple plant polysaccharides have been reported to improve MAFLD through distinct regulatory pathways. For instance, Atractylodes macrocephala polysaccharide mainly exerts antioxidant and anti-inflammatory effects via AMPK signaling (Liu et al., 2022; Zheng et al., 2025), while Pueraria lobata polysaccharide regulates gut flora, SCFAs, and bile acid metabolism but has rarely been reported to modulate tryptophan metabolism (Wu et al., 2026; Li et al., 2023). Other Dendrobium-derived polysaccharides also exhibit mild hepatoprotective activities (Fan et al., 2023; Su et al., 2023). The distinct monosaccharide composition and glycosidic linkages of DOP may account for its unique pharmacological properties. Collectively, DOP is speculated to possess a specific regulatory pattern on the tryptophan-kynurenine pathway, offering a potential novel mechanism for natural polysaccharides against MAFLD. Notably, these inferences remain preliminary without rigorous functional validation.

Collectively, this study explored the multi-target protective effects of DOP against MAFLD in SD rats based on gut-liver axis theory, providing experimental evidence for the further development of DO as a medicinal and edible resource for metabolic liver disease intervention. Nevertheless, several limitations still exist in the present study. First, the relevant conclusions are mainly derived from correlation analysis of multi-omics data, while fecal microbiota transplantation and antibiotic intervention experiments were not performed. The causal relationship between differential flora, metabolites, and phenotypic improvement remains unclear. Second, the specific functions of two key differential metabolites (ASP-ASP and formyl-5-hydroxykynurenamine) were not individually verified, and whether the TLR4/NF-κB pathway is indispensable for DOP-mediated protection needs further genetic or pharmacological validation. Third, due to experimental arrangement, bulk DOP (≥80% purity) was used for long-term animal gavage owing to large dosage demand, while high-purity DOP reference (≥85%) was adopted for *in vitro* cell experiments to reduce impurity interference. The physicochemical differences between the two batches of DOP were not systematically characterized, and potential structural disparities may cause variations in biological activity. Future studies will unify DOP raw materials and complete parallel physicochemical characterization to standardize experimental conditions. In addition, follow-up genetic intervention experiments are required to further clarify the regulatory mechanism of DOP on gut microbiota-mediated tryptophan metabolism in MAFLD.

## 5 Conclusion

This study verifies that DOP exerts prominent protective effects on MAFLD both *in vivo* and *in vitro*. Correlative multi-omics and molecular assays reveal that DOP intervention is accompanied by restored intestinal flora homeostasis, normalized intestinal metabolite profiles, recovered intestinal barrier function and attenuated TLR4/NF-κB inflammatory signaling (Figure 11). Consistent with *in vivo* outcomes, *in vitro* cellular experiments further imply that DOP could inhibit the TLR4/NF-κB inflammatory signaling in hepatocytes. These coordinated alterations provide correlative clues supporting the gut-liver axis as a potential pathway through which DOP alleviates hepatic steatosis and inflammation. Definitive causal relationships among these regulatory events still require validation via further functional experiments.

**FIGURE 11:**
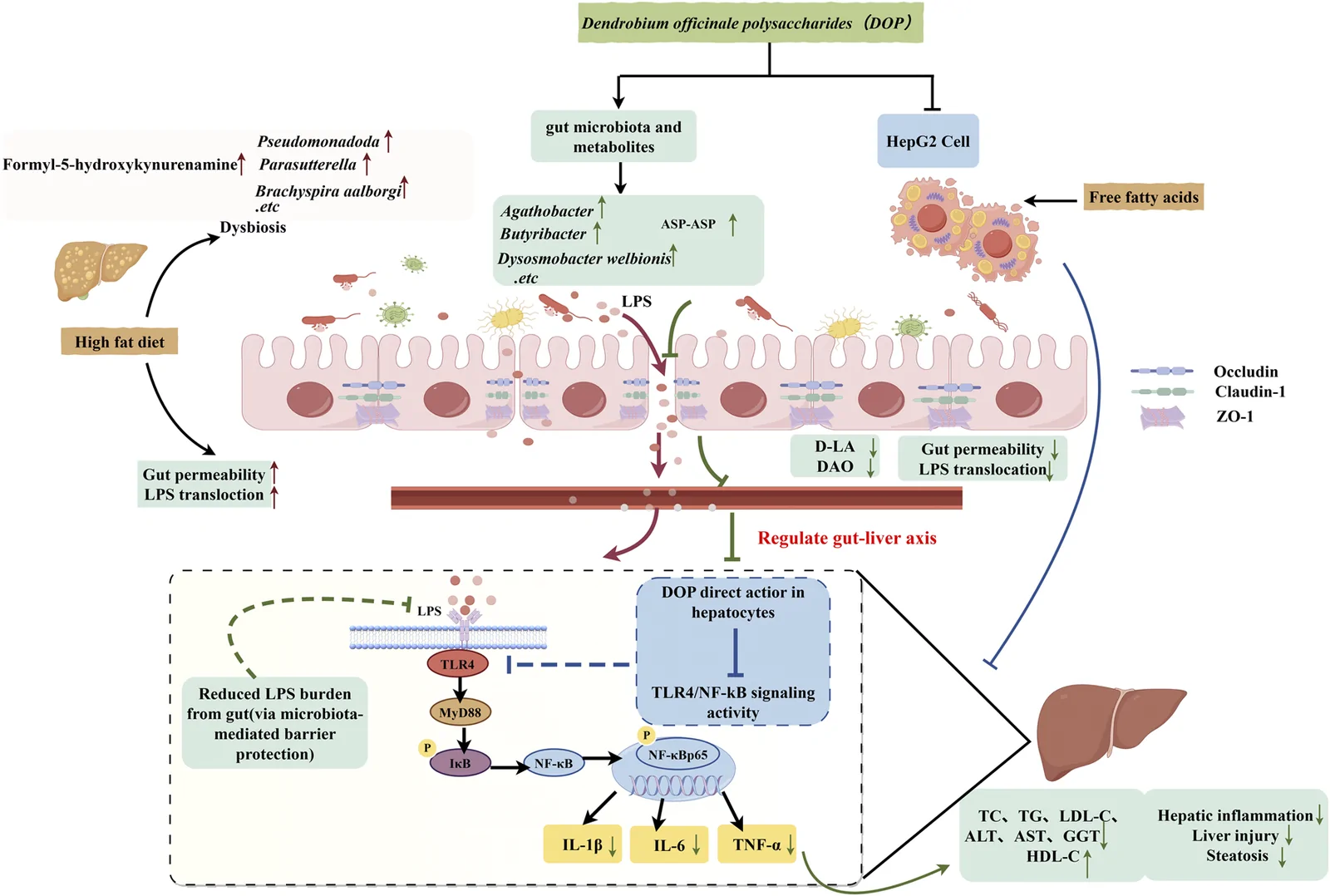
Schematic illustration depicting the protective mechanism exerted by DOP against MAFLD progression.

## Data Availability

The original contributions presented in the study are publicly available. This data can be found here: https://www.ncbi.nlm.nih.gov/bioproject/PRJNA1479921/.
