# Supplementary material for: Hepatoprotective action of Dendrobium officinale polysaccharide against MAFLD: modulation of gut microbiota-metabolite cascade controls TLR4/NF-κB inflammatory response
Source: Front Pharmacol. 2026 Sep 24;17:1953606. doi: 10.3389/fphar.2026.1953606 (PMC13645553; doi:10.3389/fphar.2026.1953606)
Supplement: Supplementary file 1 [file DataSheet1.pdf]

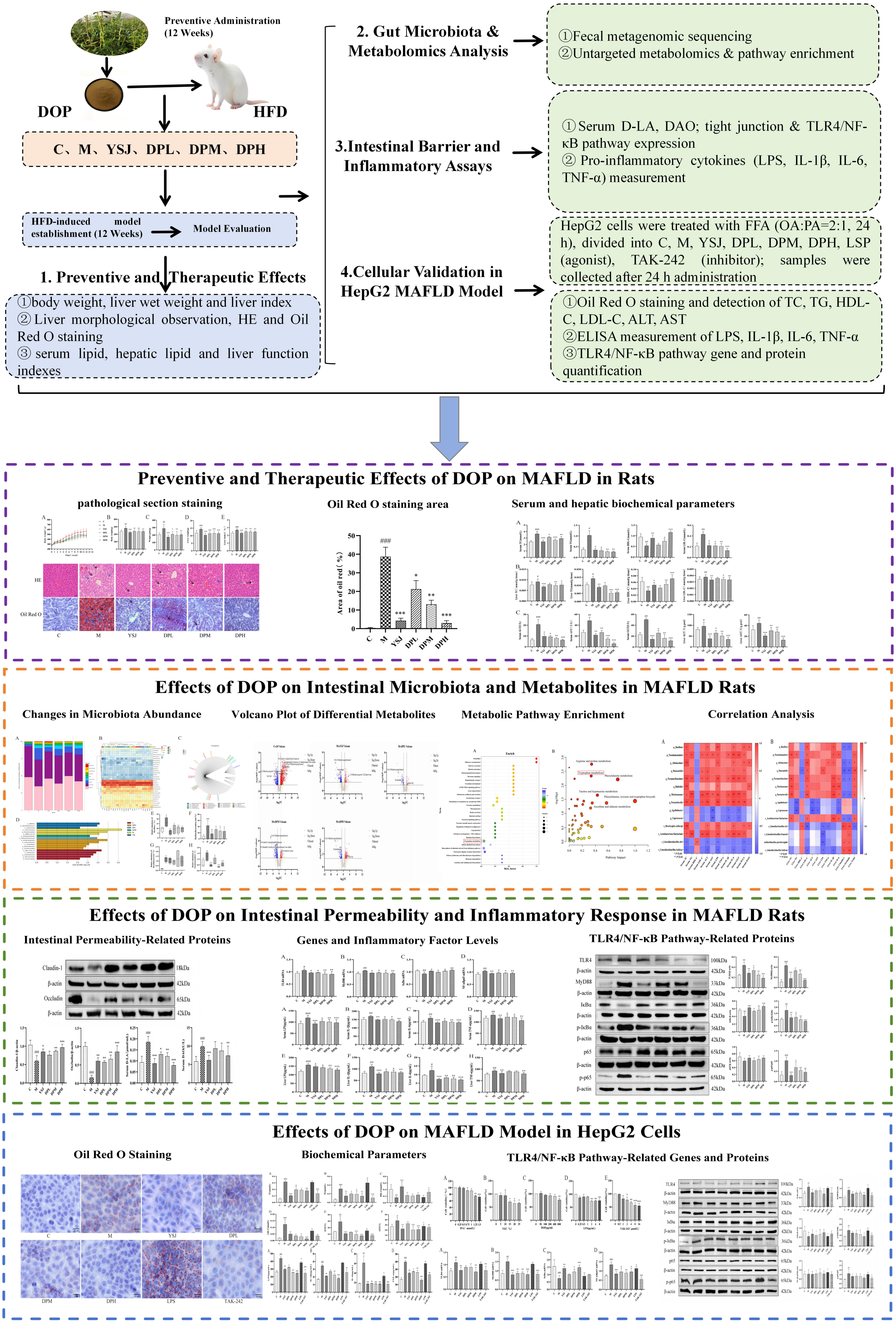

Preventive Administration (12 Weeks)
DOP
HFD
C、M、YSJ、DPL、DPM、DPH
HFD-induced model establishment (12 Weeks)
Model Evaluation
1. Preventive and Therapeutic Effects
①body weight, liver wet weight and liver index
② Liver morphological observation, HE and Oil Red O staining
③ serum lipid, hepatic lipid and liver function indexes
2. Gut Microbiota & Metabolomics Analysis
①Fecal metagenomic sequencing
②Untargeted metabolomics & pathway enrichment
3.Intestinal Barrier and Inflammatory Assays
① Serum D-LA, DAO; tight junction & TLR4/NF-κB pathway expression
② Pro-inflammatory cytokines (LPS, IL-1β, IL-6, TNF-α) measurement
4.Cellular Validation in HepG2 MAFLD Model
HepG2 cells were treated with FFA (OA:PA=2:1, 24 h), divided into C, M, YSJ, DPL, DPM, DPH, LSP (agonist), TAK-242 (inhibitor); samples were collected after 24 h administration
①Oil Red O staining and detection of TC, TG, HDL-C, LDL-C, ALT, AST
②ELISA measurement of LPS, IL-1β, IL-6, TNF-α
③TLR4/NF-κB pathway gene and protein quantification
Preventive and Therapeutic Effects of DOP on MAFLD in Rats
pathological section staining
Oil Red O staining area
Serum and hepatic biochemical parameters
Effects of DOP on Intestinal Microbiota and Metabolites in MAFLD Rats
Changes in Microbiota Abundance
Volcano Plot of Differential Metabolites
Metabolic Pathway Enrichment
Correlation Analysis
Effects of DOP on Intestinal Permeability and Inflammatory Response in MAFLD Rats
Intestinal Permeability-Related Proteins
Genes and Inflammatory Factor Levels
TLR4/NF-κB Pathway-Related Proteins
Effects of DOP on MAFLD Model in HepG2 Cells
Oil Red O Staining
Biochemical Parameters
TLR4/NF-κB Pathway-Related Genes and Proteins
